# Multidisciplinary Treatment of Post-radiation Sarcoma in the Left Axilla Following Radiotherapy for Breast Cancer: A Case Report and Review of the Literature

**DOI:** 10.7759/cureus.110740

**Published:** 2026-06-12

**Authors:** Christos Doudakmanis, Konstantinos Bouliaris, Alexis Terras, Stefania Kokkali, Georgios Agrogiannis, Stylianos Kykalos, Gerasimos Tsourouflis

**Affiliations:** 1 2nd Propaedeutic Department of Surgery, Laiko General Hospital of Athens, Athens, GRC; 2 Department of Surgery, University General Hospital of Larissa, Larissa, GRC; 3 Department of Medical Oncology, Athens General Hospital Hippocrates, Athens, GRC; 4 Department of Pathology, National and Kapodistrian University of Athens, Athens, GRC

**Keywords:** breast cancer, chest wall reconstruction, post-radiation sarcoma, radiation-induced sarcoma, radiotherapy, surgical oncology

## Abstract

Post-radiation sarcoma is a rare clinical entity, but it is considered an aggressive late complication of radiotherapy administered as adjuvant therapy for breast cancer treatment. Although radiotherapy significantly improves local disease control and survival, radiation-induced malignancies may occur years after treatment completion. Surgical resection with negative margins remains the cornerstone of management. However, treatment is often challenging because of prior irradiation, local tissue fibrosis, and advanced local disease.

We report the case of a 65-year-old woman previously treated for breast cancer with breast-conserving surgery followed by adjuvant chemotherapy and radiotherapy. Twenty-three years after completion of radiotherapy, the patient developed a progressively enlarging mass within the irradiated field. Imaging studies revealed a heterogeneous soft tissue lesion involving the chest wall. Core needle biopsy demonstrated leiomyosarcoma. Following multidisciplinary team discussion, three weeks after the core biopsy, the patient underwent wide en bloc surgical resection with chest wall reconstruction. Histopathological examination of the surgical specimen revealed high-grade undifferentiated pleomorphic sarcoma with negative surgical margins (R0 resection). Postoperative recovery was uneventful. Then the patient received adjuvant chemotherapy and radiotherapy. The patient remains disease-free after three years of follow-up.

Post-radiation sarcoma following breast cancer radiotherapy remains a rare and highly aggressive malignancy associated with poor prognosis. Early recognition and aggressive multidisciplinary treatment, particularly complete surgical excision with negative margins, are critical for improving outcomes.

## Introduction

Radiotherapy is an essential treatment modality of breast cancer treatment, especially in breast conservation surgery, as it significantly reduces local recurrence and breast cancer-related mortality. However, in some rare cases, exposure to ionizing radiation may result in secondary malignancies, including post-radiation sarcomas. Patients with breast cancer treated with radiation have 18% higher risk of developing a secondary malignancy [1]. An early original study by Taghian et al. revealed that this risk for subsequent neoplasms and, most importantly, radiation-induced sarcomas increases as the latency period increases. That being said, the incidence is 0.2% in the first 10-year period following radiotherapy and reaches up to 0.78% 30 years after receiving radiotherapy [2].

The diagnostic criteria for radiation-induced sarcoma were initially described by Cahan et al. in 1948 and include prior radiotherapy exposure, development of sarcoma within the irradiated field, histological distinction from the primary tumor, and a sufficient latency period between irradiation and sarcoma development, which usually is at least three to five years. Clinical presentation most often includes an enlarging palpable mass in the previously irradiated field. It is usually necessary to investigate the possibility of local recurrence of primary disease. In this manner, histological evaluation is required to diagnose post-radiation sarcomas [3].

Radiation-induced sarcomas arising after breast cancer treatment are biologically aggressive tumors associated with increased risk of local recurrence rates, poor disease-free survival, and poor overall survival. There is a wide variety of histological subtypes. The most common of them include undifferentiated pleomorphic sarcoma, leiomyosarcoma, angiosarcoma, osteosarcoma, malignant peripheral nerve sheath tumor, myxofibrosarcoma, and rhabdomyosarcoma [4].

Although radical surgical resection with negative margins is the cornerstone for treatment of radiation-induced sarcomas, surgery without further modalities was not associated with optimal results in terms of overall survival and local recurrence rates, highlighting the need for multidisciplinary therapy. Chemotherapy and radiotherapy could support treatment modalities, but their use independently is not curative. Positive margins are associated with significantly higher risk for local recurrence. R0 resections resulted in higher survival rates than either R1 or R2 resections. Resection margins of 2-4 cm are considered appropriate for proper disease control. In extreme cases of difficult resections, 1 cm is still considered sufficient. Therefore, negative resection margins are considered of utmost importance for prognosis [5].

Herein, we present a case of post-radiation sarcoma arising after breast cancer radiotherapy and discuss the diagnostic and therapeutic challenges associated with this rare entity. The purpose of this study is to present the therapeutic approach which included surgical resection with thoracic wall reconstruction, followed by chemotherapy and re-irradiation of the area.

This case was presented as a poster at the 43rd European Society of Surgical Oncology (ESSO) Congress at Antwerp, October 2-4, 2024.

## Case presentation

A 65-year-old female patient presented to the 2nd Propaedeutic Department of Surgery, Laiko General Hospital of Athens in March 2022 to investigate a sizeable, palpable soft-tissue mass fixed in the left axilla. She had a history of breast cancer located on the left breast, treated with breast conservation surgery and axillary lymph nodes dissection followed by adjuvant chemotherapy and radiotherapy 23 years prior to admission. The patient had remained disease-free until she noticed a rapidly enlarging lesion within the previously irradiated field. Physical examination revealed a firm, irregular mass. The mass was located in proximity to the scar tissue of the prior lymph node dissection. Imaging modalities showed a large, solid, well-defined mass in contact with the thoracic wall. It was considered close contact with the fourth rib, and contact of the tumor with the pleura could not be excluded. These findings were compatible with potential malignant necrotic enlarged lymph node, which should be excised. Figure 1 presents the preoperative imaging. 

**Figure 1:**
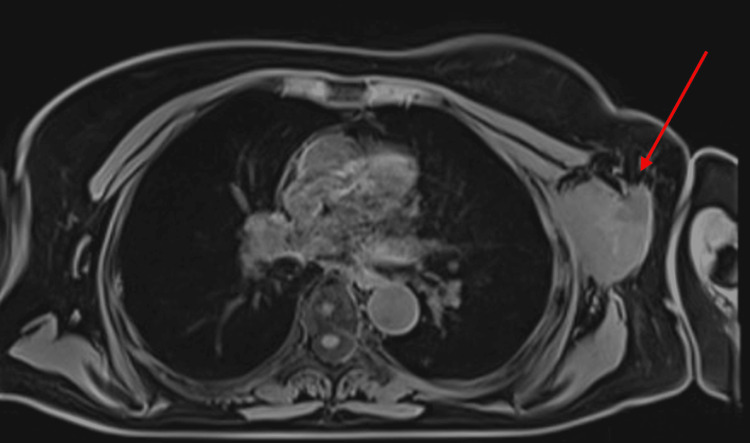
Preoperative MRI Tumor in the left axilla is shown with the red arrow. The close relation with the ribs is also visible.

Subsequently, the patient underwent a surgical biopsy of the mass for further assessment, prior to surgical excision. Histopathological results revealed findings compatible with leiomyosarcoma of the left thoracic wall, with pleiomorphic structure and tumor grade three.

Following the biopsy result and considering the localized nature of the disease and the absence of distant metastases, the Cancer Multidisciplinary Team suggested surgical resection of the mass with curative intent.

The patient underwent a scheduled operation under general anesthesia, during which a wide resection of the mass located in the left thoracic wall was performed (Figures 2, 3).

**Figure 2:**
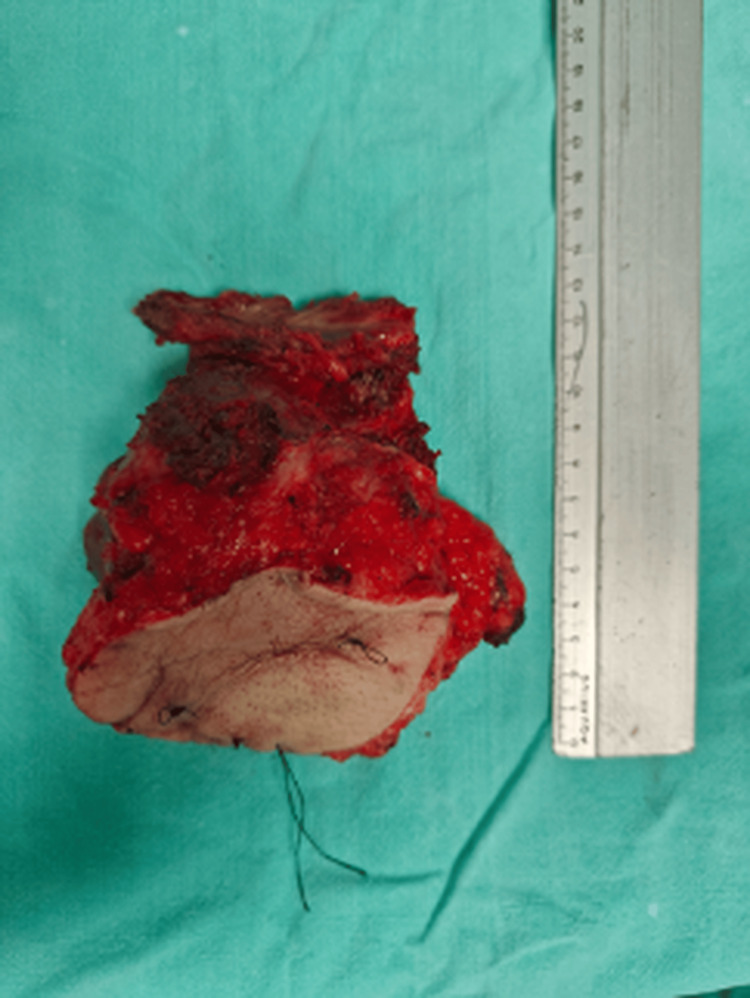
Surgical specimen At the bottom part of the specimen with the suture the skin with the subcutaneous tissue are shown, while at the top part of the specimen the fourth rib with the parietal pleura are shown.

**Figure 3:**
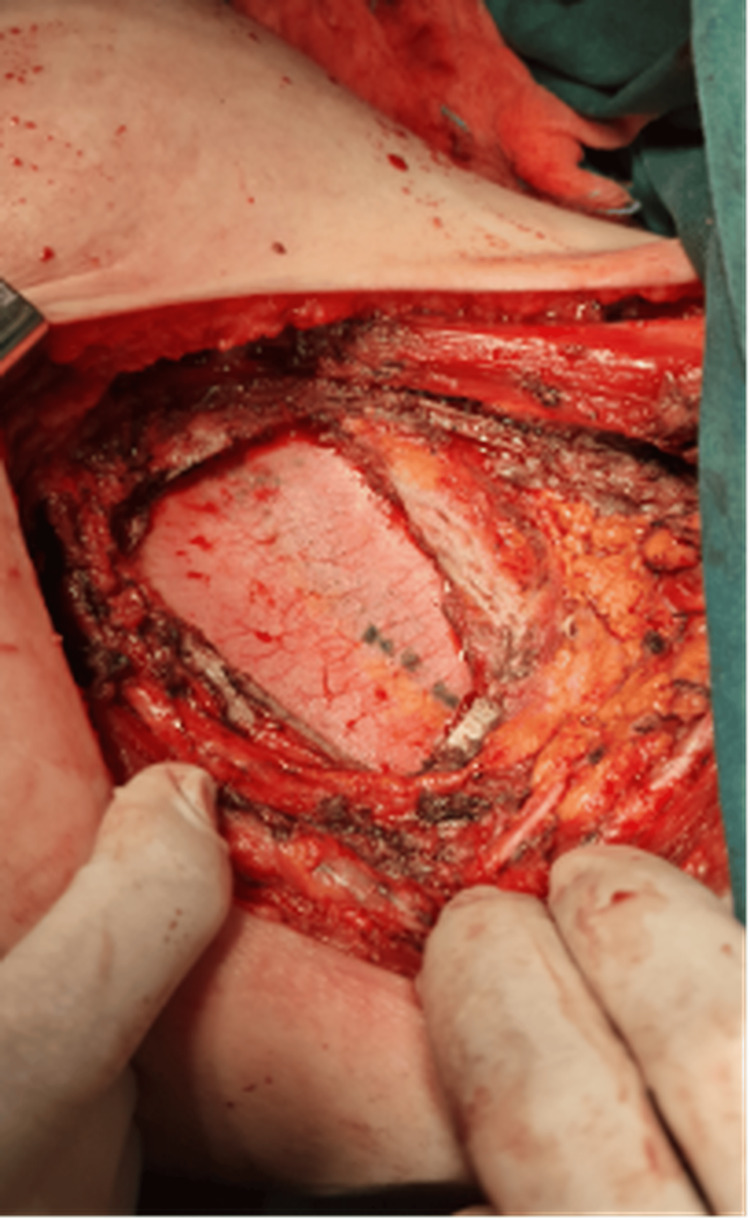
Surgical field following resection Part of the fourth rib is removed and splachnic pleura is shown.

A part of the fourth rib was also resected due to infiltration from the tumor, and the thoracic wall defect was repaired using synthetic polypropylene mesh and the defect of soft-tissue was reconstructed using a vascularized myocutaneous flap from the left latissimus dorsi muscle (Figures 4, 5).

**Figure 4:**
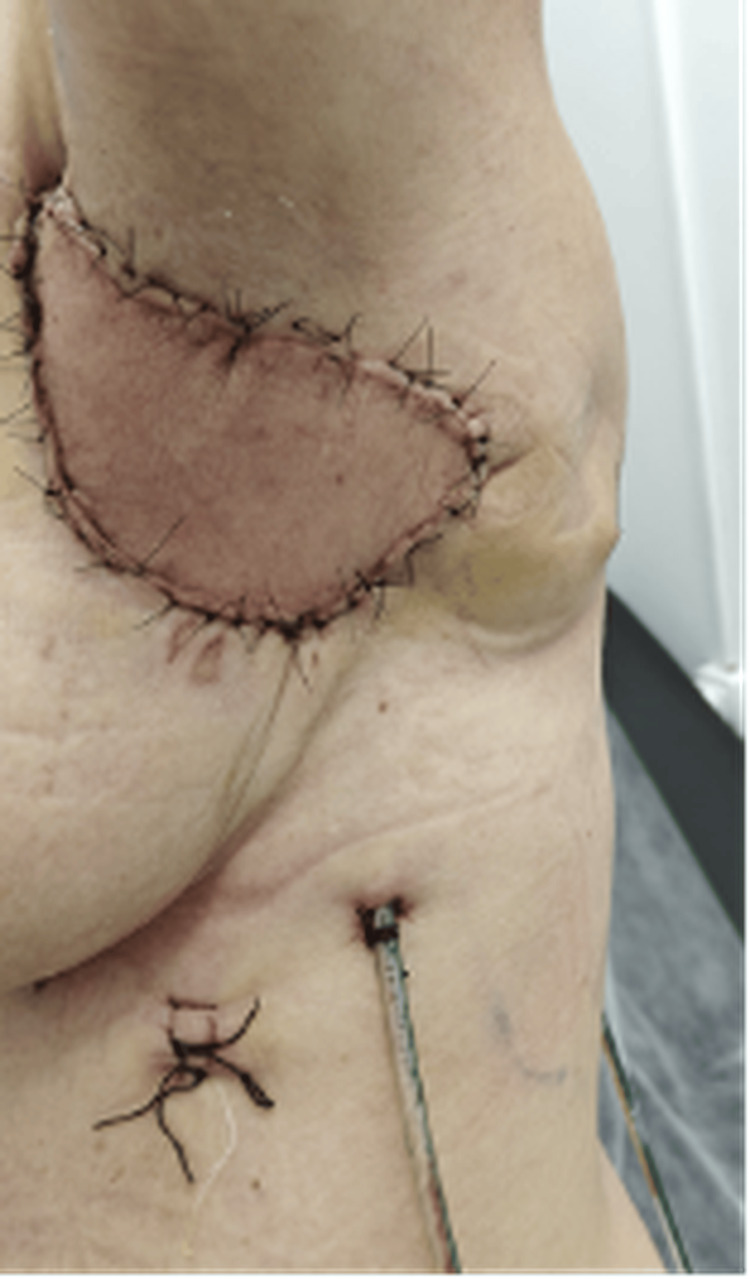
Postoperative image of the chest wall reconstruction using polypropylene mesh and myocutaneous latissimus dorsi (LD) flap Left axilla is presented following the complex reconstruction. The flap is in place and the presence of a negative-pressure drain is noted.

**Figure 5:**
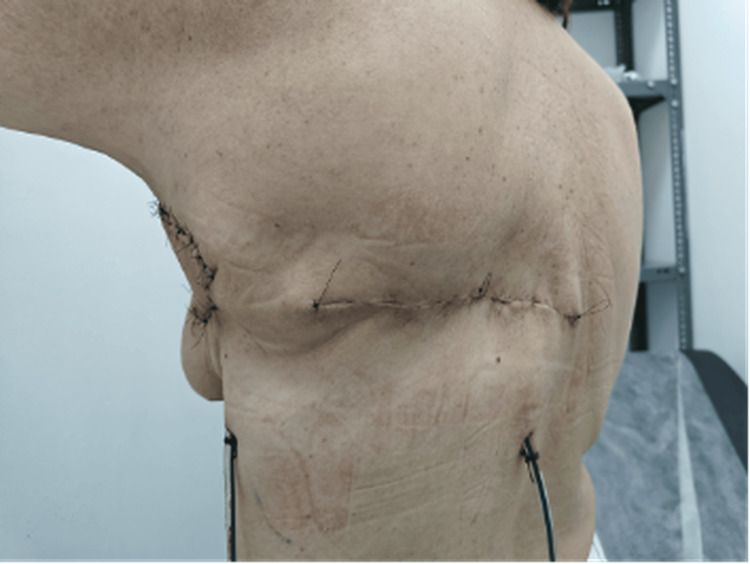
Postoperative of image of the donor area of the myocutaneous latissimus dorsi (LD) flap The donor area of the myocutaneous LD flap is shown. No large defects were present. Surgical drains were still in place.

The postoperative course was uneventful, and the patient was discharged on postoperative day five. 

Pathology examination of the specimen showed an undifferentiated pleiomorphic sarcoma of the thoracic wall, tumor grade three, with negative surgical margins (R0 resection) by more than 1 cm. Then, the patient was referred to the Department of Oncology for adjuvant chemotherapy and radiotherapy, based on the decision of the Cancer Multidisciplinary Team. The patient successfully received six cycles of doxorubicin-based chemotherapy. Despite the radical resection, the patient also received radiotherapy, as it was considered high-risk due to the difficulties of surgical resection in case of local recurrence.

At this point, at the yearly follow up, four years following the surgical resection and complex reconstruction, the patient is still disease-free, with no evidence of local recurrence or distant metastasis.

## Discussion

Radiotherapy is a known potent carcinogen, and it is considered a side effect of cancer treatment. Usually, latent periods from exposure to secondary neoplasia are a few years to a few decades. Breast cancer patients who receive radiotherapy treatment are at a long-term risk for secondary cancer incidence, as a side effect of their initial treatment. Usually, this mainly affects patients who undergo breast-conserving surgeries and, less commonly, patients who have had radiotherapy for chest wall infiltration following radical mastectomy. While incidence rates are generally low, there is a gradient increase from 0.2% at 10 years to 0.78% [6].

The pathogenesis of post-radiation sarcomas is associated with radiation-induced genomic instability and DNA damage, leading to malignant mesenchymal transformation [7]. Diagnosis may be delayed because early lesions can mimic benign post-radiation changes, fibrosis, or local recurrence of breast cancer. Consequently, clinicians should maintain a high index of suspicion for any new enlarging lesion arising within a previously irradiated field. These sarcomas usually present as a new mass lesion within the previously irradiated field. Imaging modalities do not show specific pathognomonic features and can be difficult to interpret.

As in our case, radiotherapy for breast cancer is one of the most common causes of post-radiation sarcomas, and one should investigate the risk of local recurrence of the primary tumor. Local recurrence of breast cancer may be difficult to exclude from imaging. Diagnosis of these soft-tissue tumors requires core biopsy in order to distinguish sarcoma from local recurrence. However, as shown in our case, core biopsy comes with certain limitations. The most important are the small sample size, which could lead to inconclusive results, and the fact that in large tumors, only a portion of them is examined. That being said, certain tumors like sarcomas are characterized by vast heterogeneity. Larger tumors and the higher grade of the sarcoma are independent factors associated with poorer prognosis. Anatomical sites of incidence, like the retroperitoneum and head and neck, are also associated with poor prognosis, as radical surgical treatment is challenging [8].

Complete surgical resection with negative margins remains the cornerstone of treatment and represents the most important prognostic factor for survival. Optimal resection results in five-year survival rates above 50%. Survival rates are significantly lower in patients with metastatic disease or with local recurrence of the sarcoma despite radical surgical treatment. Large tumors are associated with poor prognosis. Low-grade tumors are correlated with better outcomes. Due to prior irradiation and extensive local tissue damage, surgery is frequently technically demanding and may require complex chest wall reconstruction and multidisciplinary management [9]. In our case, en bloc resection of the tumor with part of the fourth rib and parietal pleura. In this context, adjuvant chemotherapy or radiotherapy may be required. However, independent administration of these modalities is not associated with optimal outcomes. The combination of these treatments is considered the best approach to date [10].

However, there is insufficient evidence to propose a standard of care. Adjuvant chemotherapy remains controversial because of the rarity and histological heterogeneity of radiation-induced sarcoma. Chemotherapy and radiotherapy provide a potential benefit in high-grade or large tumors and in highly selected patients [11]. Omission of surgical resection should not be considered, as patients who receive only chemotherapy or radiotherapy have significantly poorer outcomes in terms of survival rates [12]. Re-irradation is not suggested routinely. It is considered an option in selected cases of post-radiation sarcoma that either have positive margins or residual disease following resection, or in recurrent cases where further resection is not possible or difficult, like in our case. There are no large scale studies reported in the literature. Only selected cases or case series where re-irradiation was chosen are reported. In most of these cases, the sarcomas were found on the extremities or in the retroperitoneal space [9]. 

Our case highlights the importance of early diagnosis and aggressive multidisciplinary management in achieving favorable oncological outcomes in patients with post-radiation sarcoma after breast cancer treatment.

## Conclusions

Soft-tissue sarcomas are rare but worrisome features following radiotherapy for breast cancer. More specifically, post-radiation sarcoma following breast cancer radiotherapy is a rare but highly aggressive secondary malignancy. Depending on the preoperative diagnosis through biopsy, surgical resection is often indicated. However, complex resections may be required. We presented a case from our department in which radical resection of the tumor en bloc with part of the fourth rib and parietal pleura and chest wall reconstruction with latissimus dorsi flap were performed. In these complex cases, a multi-disciplinary approach is necessary as the use of meshes and flaps is often needed. Also, a multimodal approach is required in these cases, and treatment should be highly personalized, as due to the rarity and heterogeneity of the disease, there is no standardized approach. Our patient received six cycles of doxorubicin-based chemotherapy postoperatively. She also underwent re-irradiation of the already irradiated area, as she was considered high-risk due to the inability of further surgical resection in case of local recurrence. 
